# Triple Abdominal Vascular Compression in a Young Woman: A Case Report of the Simultaneous Presentation of Superior Mesenteric Artery, Nutcracker, and May-Thurner Syndromes

**DOI:** 10.7759/cureus.94680

**Published:** 2025-10-15

**Authors:** JC Hurtado-Tobar, JS Chacon-Gonzalez, Silvana Galezo-Cuevas, K Portela-Buelvas, Jairo J Tejera-Hernández

**Affiliations:** 1 Department of General Surgery, Integrated Health Services Network – Hospital Occidente de Kennedy, Bogotá, COL; 2 Faculty of Medicine, National University of Colombia, Bogotá, COL; 3 Department of Hospitalization, Hospital Occidente de Kennedy, Bogotá, COL; 4 Department of Internal Medicine, Intermedy, Health Services Provider Institution (IPS), Medellín, COL

**Keywords:** abdominal pain, computed tomography angiography, may-thurner syndrome, renal nutcracker syndrome, superior mesenteric artery syndrome

## Abstract

Multiple vascular compression of the abdomen is a rare condition characterized by the coexistence of anatomically independent but clinically related syndromes. This report presents the case of a young woman with chronic digestive symptoms, food intolerance, significant unintentional weight loss, and postprandial vomiting, in whom the presence of superior mesenteric artery syndrome (Wilkie syndrome), nutcracker syndrome, and May-Thurner syndrome was confirmed by angiotomography. The combination of findings represents an exceptional form, with overlapping manifestations that make early recognition difficult. Management was conservative, with a nutritional approach and clinical follow-up achieving a partial improvement of symptoms. This case highlights the need to consider uncommon vascular diagnoses in patients with persistent abdominal pain, especially when indirect clinical signs are present and emphasizes the value of advanced imaging studies in the diagnosis of vascular disorders.

## Introduction

Abdominal vascular compression syndromes, such as superior mesenteric artery syndrome (Wilkie syndrome), nutcracker syndrome, and May-Thurner syndrome, result from a reduction of the aortomesenteric angle that leads to an extrinsic compression of retroperitoneal vessels and organs [1,2]. These entities share predisposing factors such as rapid weight loss, hypercatabolic states, eating disorders, and recent abdominal or spinal surgery, all of which cause loss of retroperitoneal fat and promote vascular narrowing [1].

Although they share a common pathophysiological mechanism, each syndrome affects different structures and produces distinct clinical manifestations. In Wilkie syndrome, compression of the third portion of the duodenum causes postprandial abdominal pain, nausea, vomiting, early satiety, and weight loss [3]. Nutcracker syndrome involves compression of the left renal vein, leading to hematuria, proteinuria, flank pain, or pelvic congestion [1,4]. In contrast, May-Thurner syndrome results from compression of the left iliac vein by the right common iliac artery, presenting with leg pain, edema, deep vein thrombosis, or signs of chronic venous insufficiency [5,6].

Because symptoms often overlap and remain nonspecific, diagnosis can be challenging. Computed tomography with vascular reconstruction is essential for identifying these syndromes and excluding alternative causes [7,8].

We report the case of a patient with chronic abdominal pain and imaging findings consistent with triple vascular compression, in whom the simultaneous presence of Wilkie, nutcracker, and May-Thurner syndromes was documented after more than 12 months of symptom progression.

## Case presentation

We report the case of a 25-year-old woman with no relevant surgical, gynecological, or family history who consulted on multiple occasions since August 2023 for persistent abdominal pain, which was oppressive and high intensity on the visual analog pain scale (7/10), with variable location, but predominantly in the epigastrium and mesogastrium, which subsided with the use of analgesics. In 2024, these symptoms were intermittent and associated with nausea, frequent vomiting, and hyporexia. The patient reported a feeling of early satiety, abdominal distension after meals, and fatigue. The symptoms intensified after eating and were sometimes partially relieved in the left lateral decubitus position, although this finding went unnoticed in the initial consultations.

In early 2024, she was evaluated at various institutions. Presumptive diagnoses of uncomplicated urinary tract infection, functional dyspepsia, pubalgia, and subacute appendicitis were documented, without high-resolution imaging studies, so the clinical approach initially focused on these causes. Multiple laboratory tests were ordered, which showed no significant abnormalities, with normal liver and kidney function and blood counts initially within normal limits (Table 1). Therefore, the patient was treated symptomatically with analgesics, antispasmodics, and antibiotics, with improvement and recurrence of symptoms.

**Table 1:** Laboratory test results MCV: Mean corpuscular volume; MCH: mean corpuscular hemoglobin; Ca: calcium; Na: sodium; K: potassium; P: phosphorus; BUN: blood urea nitrogen; HIV: human immunodeficiency virus; TSH: thyroid-stimulating hormone; N/A: not applicable.

Test	Results	Reference values
Hematology		
Leukocytes	4,020 μL	4,500-11,000 μL
Neutrophils	1,840 μL	1,800-7,500 μL
Lymphocytes	1,540 μL	1,200-3,400 μL
Hemoglobin	11.7 g/dL	12-16 g/dL
Hematocrit	36%	36-46%
MCV	85.9 fL	80-100 fL
MCH	27.90 pg	27-32 pg
Platelets	263,000 μL	150,000-450,000 μL
Human chorionic gonadotropin (hCG)	Negative	N/A
Blood chemistry		
Total protein	6.63 g/dL	6.0-8.3 g/dL
Albumin	4.24 g/dL	3.5-5.0 g/dL
Total cholesterol	152 mg/dL	<200 mg/dL
Triglycerides	100 mg/dL	< 150 mg/dL
Ca	8.1 mg/dL	8.5-10.5 mg/dL
Na	138.8 mEq/L	135-145 mEq/L
K	3.67 mEq/L	3.5-5.0 mEq/L
P	4.9 mEq/L	2.5-4.5 mEq/L
BUN	13.8 mg/dL	6.0-20.0 mg/dL
Creatinine	0.86 mg/dL	0.50-1.0 mg/dL
Basal blood glucose	71.5 mg/dL	70.0-110.0 mg/dL
Endocrinology		
Thyroid-stimulating hormone TSH	3.26 uUl /ml	0.27-4.20 uUl /ml
Screening for drugs of abuse		
Cocaine, amphetamines, methamphetamines, marijuana, methadone, methylenedioxymethamphetamine, morphine, barbiturates, benzodiazepines, tricyclic antidepressants, opiates, and phencyclidine.	Negative	N/A
Immunohematology		
Rh or D factor	Positive	N/A
Serology/immunology		
Toxoplasma IgG antibodies	Non-reactive	Reactive
Toxoplasma IgM antibodies	Non-reactive	Reactive
Rapid HIV test for anti-HIV-1 and anti-HIV-2 antibodies, p24 antigen	Negative	N/A
Rapid test for treponemal antibodies (IgG/IgM) against Treponema pallidum	Negative	N/A
Urinalysis		
Urine density	1.015	1.005-1.030
Urine pH	8.0	4.5-8.0
Leukocytes in urine	Negative	N/A
Nitrites in urine	Negative	N/A
Proteins in urine	Negative	N/A
Glucose in urine	Normal	N/A
Ketone bodies in urine	Negative	N/A
Urobilinogen in urine	Normal	N/A
Bilirubin	Negative	N/A
Blood	10 ery/uL	N/A
Urinary sediment: epithelial cells, bacteria, leukocytes, red blood cells	0-2XC + 0-2XC Occasional	N/A

Around October 2024 (approximately 10 months after symptom onset), she began to show signs of weight loss of about 10-12 kg from an initial weight of 59 kg (height: 179 cm), accompanied by asthenia, anorexia, and occasional episodes of bilious vomiting. Despite this, no organic structural cause was suspected until February 2025 when hospitalization was decided due to evident nutritional deterioration and incapacitating digestive symptoms.

During the in-hospital assessment, eating disorders were ruled out by psychiatric evaluation. Laboratory tests revealed mild hypochromic microcytic anemia, with no evidence of active bleeding or malabsorption, and infectious and hormonal tests showed no abnormalities.

**Figure 1:**
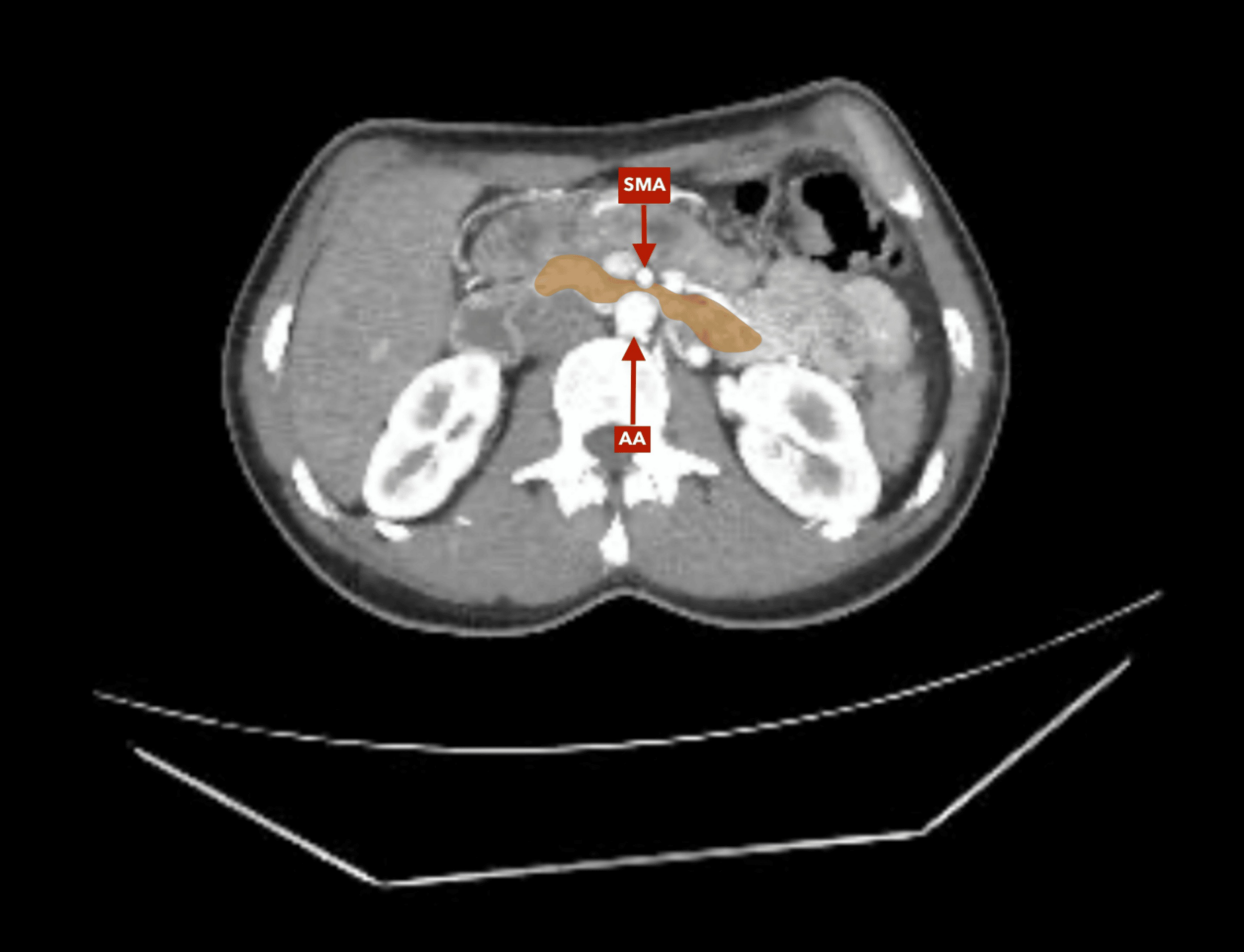
Contrast-enhanced abdominal CT scan, axial reconstruction, arterial phase. Evidence of duodenal compression by the superior mesenteric artery (SMA) on the abdominal aorta (AA). No evidence of pneumoperitoneum.

**Figure 2:**
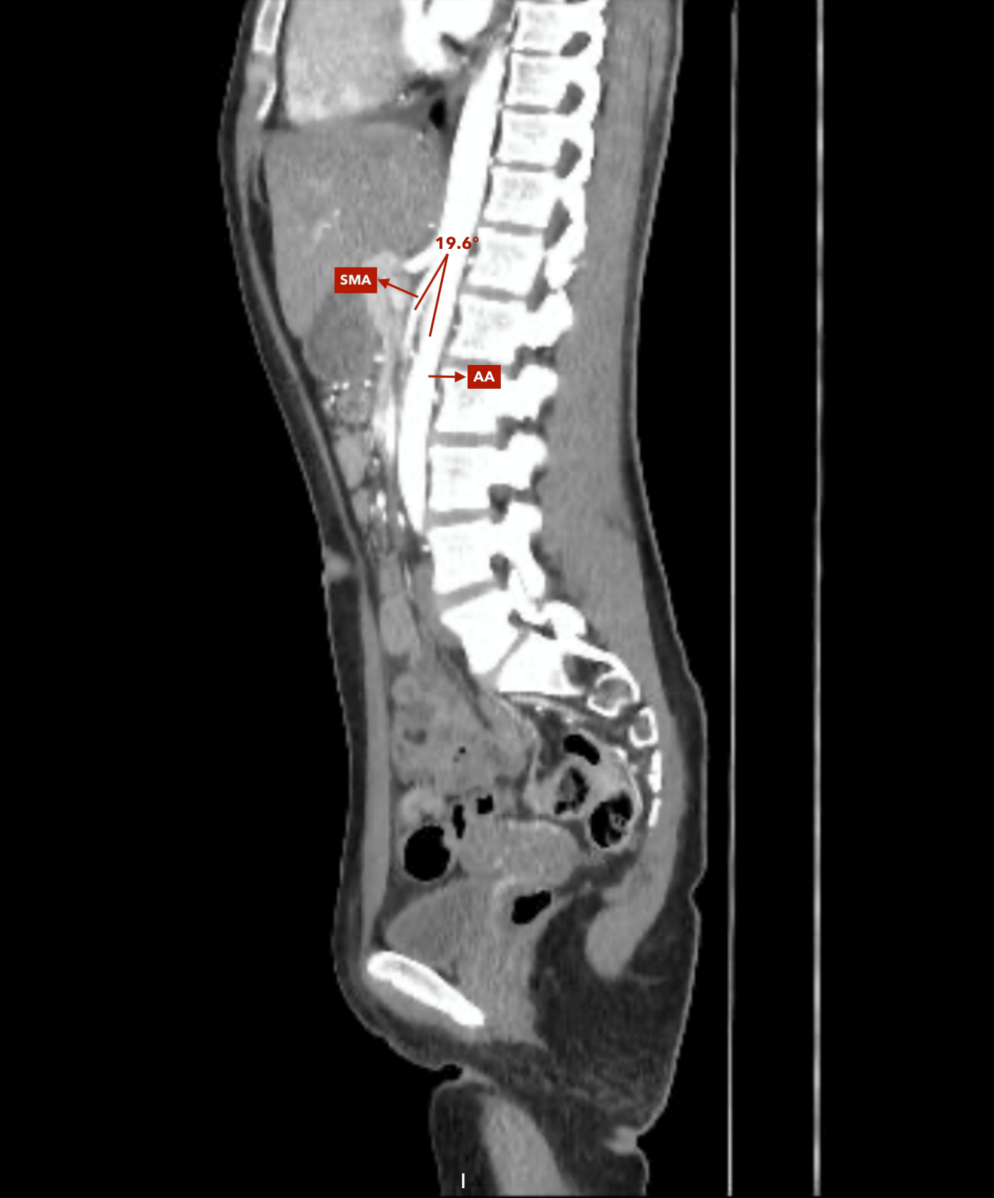
Contrast-enhanced abdominal CT scan (sagittal reconstruction, arterial phase) Approach for measuring the aorto-mesenteric angle. Angle between the abdominal aorta (AA) and superior mesenteric artery (SMA) of 19.6°.

**Figure 3:**
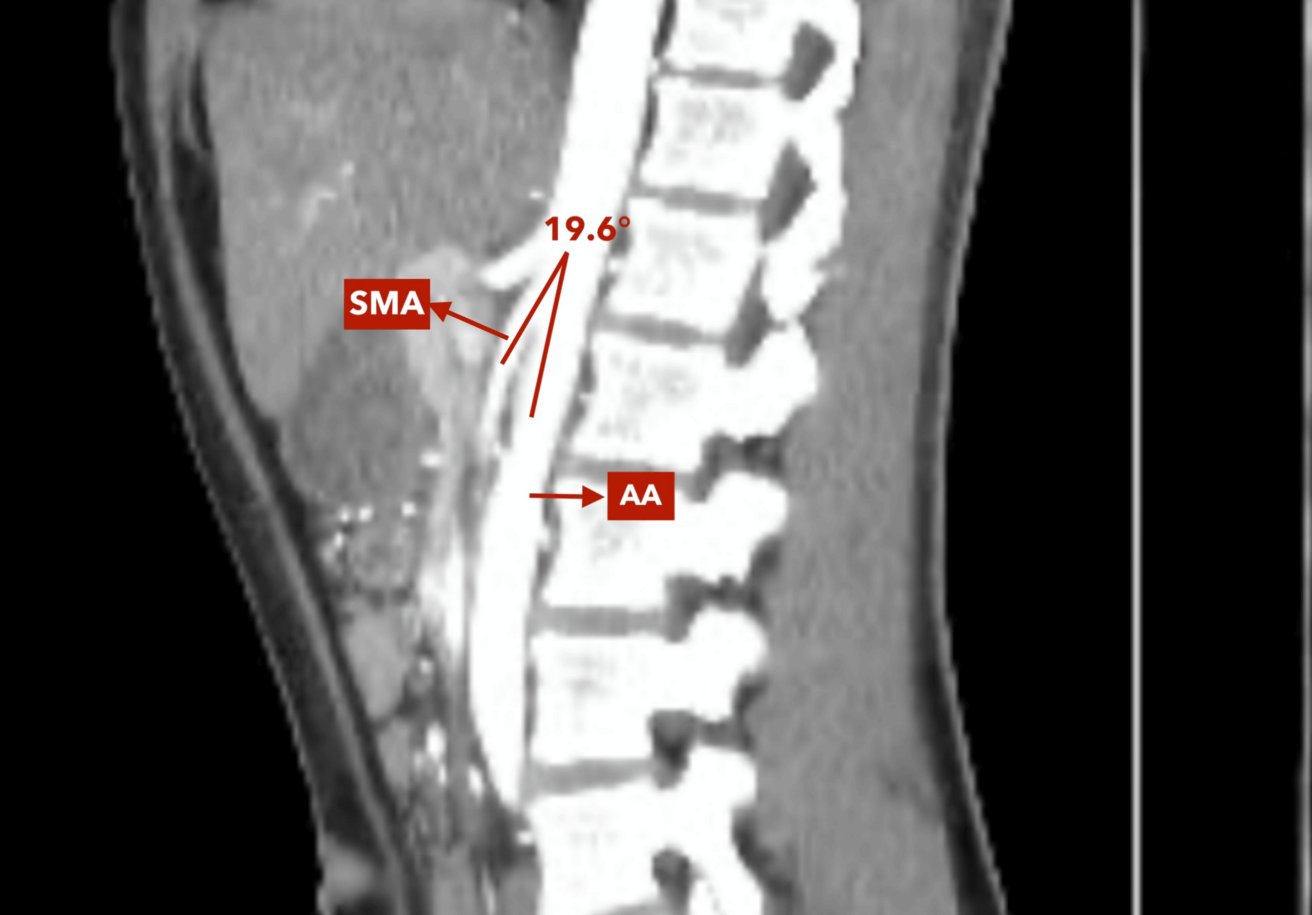
Zoomed-in sagittal reconstruction of contrast-enhanced abdominal CT scan (sagittal reconstruction, arterial phase) This image corresponds to a zoomed-in view of Figure 2, allowing closer visualization of the anatomical details and measurement of the aortomesenteric angle (19.6°) between the abdominal aorta (AA) and the superior mesenteric artery (SMA).

**Figure 4:**
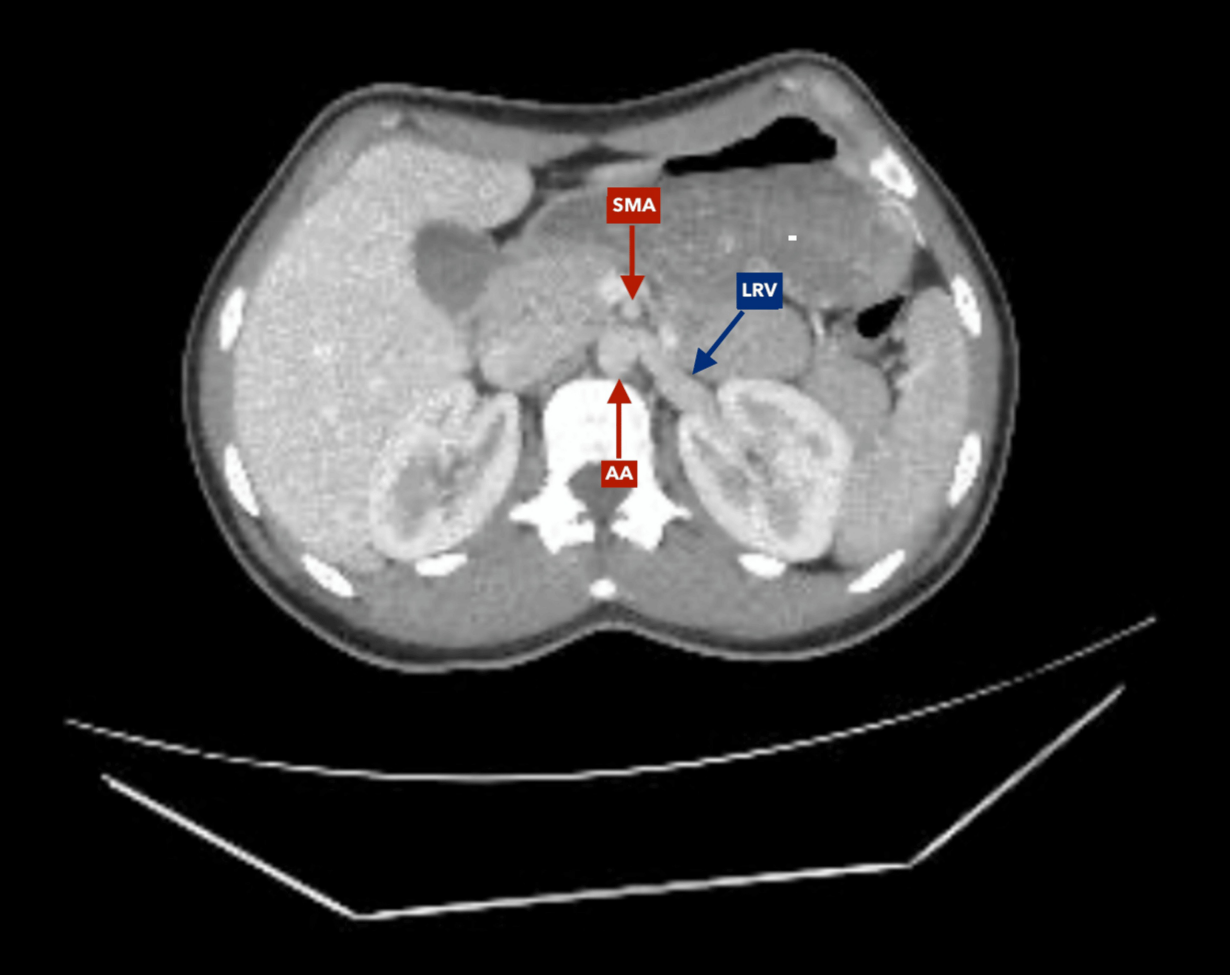
Contrast-enhanced computed tomography of the abdomen, axial reconstruction, venous phase Nutcracker syndrome. Stenosis secondary to compression of the left renal vein (LRV) by the superior mesenteric artery (SMA) over the abdominal aorta (AA).

**Figure 5:**
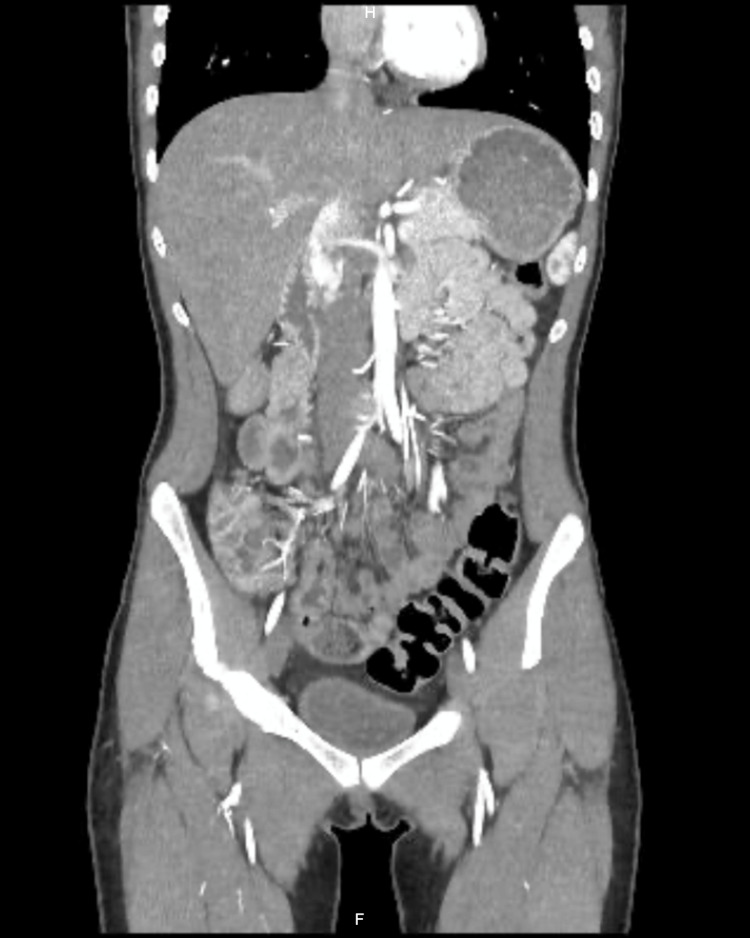
Contrast-enhanced abdominal computed tomography, coronal reconstruction, arterial phase May-Thurner syndrome. Diagram illustrating the anatomy of compression of the left common iliac vein by the right common iliac artery over the lumbar vertebral bodies

**Figure 6:**
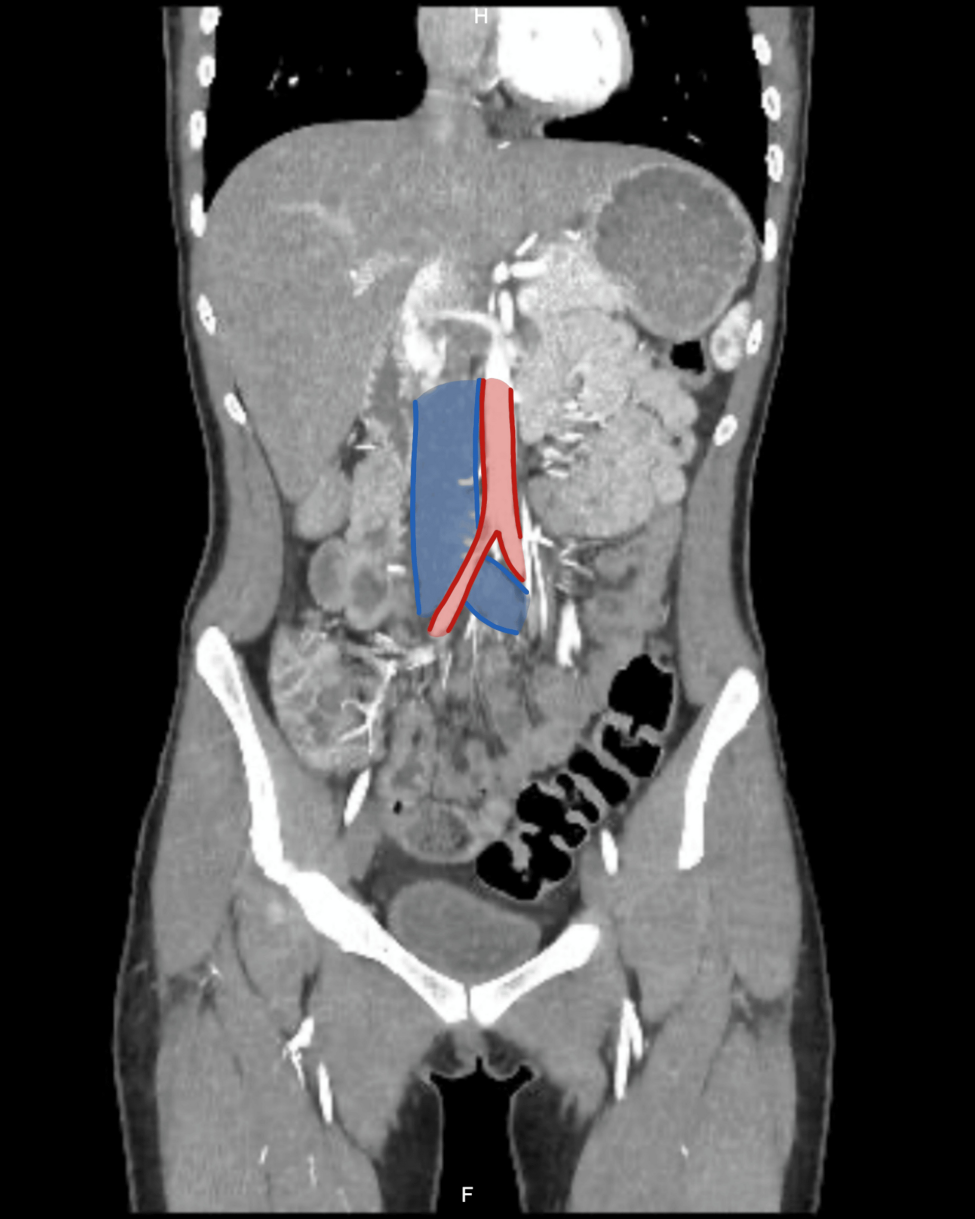
Contrast-enhanced abdominal computed tomography, coronal reconstruction, arterial phase Compression of the left common iliac vein (LCIV) by the right common iliac artery (RCIA) over the L4 vertebral body is evident. Right common iliac vein (RCIV). Left common iliac artery (LCIA).

**Figure 7:**
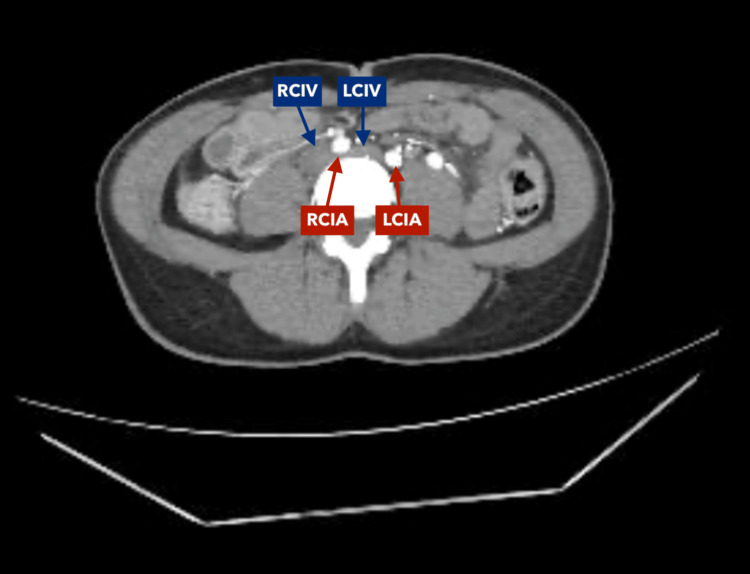
Contrast-enhanced abdominal computed tomography, coronal reconstruction, arterial phase Compression of the left common iliac vein (LCIV) by the right common iliac artery (RCIA) over the L4 vertebral body is evident. Right common iliac vein (RCIV). Left common iliac artery (LCIA).

A contrast-enhanced abdominal angiotomography with vascular reconstruction was requested due to suspicion of compression syndrome due to persistent pain. The study showed a critical reduction in the aortomesenteric angle, with compression of the third portion of the duodenum between the aorta and the superior mesenteric artery (Figures 1-3). In addition, significant compression of the left renal vein between the aorta and the superior mesenteric artery was documented, consistent with nutcracker syndrome (Figure 4), and finally, compression of the left common iliac vein by the right iliac artery against the vertebral body, a finding suggestive of May-Thurner syndrome (Figures 5-7).

These findings led to a diagnosis of triple vascular compression (Wilkie, nutcracker, and May-Thurner), an extremely rare condition, especially in young patients with no predisposing factors. Management was initially conservative, with a specialized nutritional approach that included a high-calorie, fractionated diet for eight days, left lateral decubitus position after meals, symptom control, and follow-up by vascular surgery, with surgical intervention ruled out at that time. The patient showed significant symptomatic improvement five days after starting nutritional supplementation. Nutritional support was administered orally, through a high-calorie diet divided into six meals, supplemented with a food for special medical purposes (FSMP): whey protein isolate with maltodextrin, 32 g, administered twice a day. She was discharged with scheduled outpatient follow-up for vascular surgery.

## Discussion

Vascular compression syndromes are an uncommon cause of chronic abdominal pain and their diagnosis is often delayed due to the nonspecific nature of symptoms and low clinical suspicion. Certain features, such as postprandial pain, vomiting, and progressive weight loss, should raise suspicion for these entities [9,10].

In this patient, the coexistence of three vascular compression syndromes - superior mesenteric artery syndrome, nutcracker syndrome, and May-Thurner syndrome - represents an exceptionally rare finding with significant pathophysiological implications. The marked loss of retroperitoneal and mesenteric fat likely led to a critical narrowing of the aortomesenteric angle, producing simultaneous compression of the duodenum, the left renal vein, and the left common iliac vein. This suggests a shared anatomical and mechanical mechanism rather than three independent vascular events [11].

From a diagnostic perspective, the initial evaluation appropriately ruled out common causes of chronic abdominal pain, including peptic, biliary, gynecologic, endocrine, and infectious conditions, all of which were unremarkable. Psychiatric assessment also excluded eating disorders. These negative findings, together with persistent and debilitating symptoms, justified the use of contrast-enhanced computed tomography, which revealed the triple vascular compression [12,13].

Angiotomography with vascular reconstruction remains the gold standard for diagnosis, allowing simultaneous visualization of arterial and venous structures and quantification of the aortomesenteric angle. In this case, it was essential to differentiate vascular compression from functional gastrointestinal disorders or psychosomatic causes, as the clinical presentation may overlap.

The patient responded favorably to conservative management, consisting of nutritional management and postural measures, achieving weight recovery and complete resolution of symptoms. This clinical course reinforces the concept that early nutritional support can restore mesenteric fat and relieve compression, avoiding surgical intervention in selected patients [13].

This case highlights the importance of maintaining a high index of suspicion for vascular compression syndromes in young patients with unexplained chronic abdominal pain and weight loss. It also underscores the need for an integrated diagnostic approach combining clinical evaluation, imaging, and multidisciplinary management, which is crucial for accurate diagnosis and successful outcomes.

## Conclusions

This case highlights the diagnostic and therapeutic value of early recognition of vascular compression syndromes, even in the absence of typical risk factors. The simultaneous occurrence of Wilkie, nutcracker, and May-Thurner syndromes demonstrates that shared pathophysiological mechanisms, such as loss of retroperitoneal fat and altered venous return, can coexist in young patients with chronic abdominal pain. Early implementation of conservative, nutrition-focused management can promote anatomical recovery and symptom resolution, emphasizing the need for a multidisciplinary approach that integrates clinical, nutritional, and radiological perspectives.
